# Andes virus and sin nombre virus: Similarities and distinctive features

**DOI:** 10.6026/973206300222953

**Published:** 2026-05-31

**Authors:** Francesco Chiappelli

**Affiliations:** 1 Dental Group of Sherman Oaks, Sherman Oaks, CA 91403 & UCLA Center for the Health Sciences, Los Angeles, CA 90095

**Keywords:** Immunity, Orthohantavirus (OHV), Sin Nombre Virus (SNV), Andes Virus (ANDV), hemorrhagic fever with renal syndrome (HFRS), hantavirus cardiopulmonary syndrome (HCPS), hantavirus pulmonary syndrome (HPS), Gn and GC glycoprotein moieties, nucleocapsid protein (N), beta3(β3)-integrin (CD61), antivirals, vaccine

## Abstract

The threat of hanta virus infection has come to the forefront in recent weeks, following the outbreak of the Andes virus aboard the Dutch-flagged MV Hondius cruise ship during its spring 2026 voyage. Several passengers and crew members got infected because Andes virus is the sole known hanta virus to trigger human-to-human infection. All exposed passengers have returned to their countries in Asia, the Americas and Europe, and remain in quarantine to this day because of Andes virus extended incubation period (35-45 days). Several infected patients have died due to Andes high mortality (35-50%). Taken together, the unfolding of these alarming facts has ignited fears of a new hanta virus pandemic. Here, we briefly outline certain among the major similarities and distinctive features between Andes virus and Sin Nombre virus, the two principal hanta viruses known today to infect humans. This writing emphasizes how timely and critical it is to develop concerted research and accelerated testing programs for anti-hanta virus vaccines and antivirals.

## The Hantan virus family:

US service men first reported certain viral ailments - i.e., a hemorrhagic fever with renal syndrome - while serving along the Hantan River during the Korean War (1950-53). The causative virus was eventually isolated, named Hantaan Virus [i.e., Hantaviridae, genus Orthohantavirus hantanense, OHV], and characterized as a segmented (viz, 3 segments: Large, Medium and Small) negative sense single stranded RNA (-ssRNA) virus in South Korea in 1978 [1]. Morphologically, OHV virions are roughly spherical with a quasi-symmetric icosahydral (n=12) lattice, characterized by surface spikes made up of tetramers of the glycoproteins Gn and Gc that extend from a rudimentary lipid bilayer envelope. Contained within the envelope are the virions' nucleocapsids composed of several copies of the nucleocapsid protein (N) that is encoded by the Small viral genomic segment, and which interacts with the three segments of the viral genome. The viral RNA-dependent RNA polymerase, encoded by the Large RNA segment, is found proximally. The Medium segment encodes for a glycoprotein precursor that is eventually cleaved by the hosts signal peptidase into the Gn and Gc moieties [1,2]. The replication process of OHV in host cells begins with the attachment of the spike tetramers to integrin moieties on the host cell membrane - specifically αx/beta3 (β3)-integrin (i.e., αx/CD61). Following fusion of the host's and virion's lipid bilayer, the virion is internalized, transported into clathrin- coated vesicles, and, fused, principally via the virion's Gc type II viral fusion protein, into cytoplasmic endosomes, whose low pH favors the release of the viral segmented genome and ribonucleoproteins complex into the cytoplasm. Viral reverse transcription into complementary DNA, replication, transcription and translation of new viral components commence for their encapsulation by the N proteins, assembly in the Golgi apparatus, and release of new virions by exocytosis [2, 3]. Much still remains elusive regarding the molecular mechanisms of OHV recognition by the innate immune response and viral antagonism within the host. What is clear, however, is that both Gn and Gc can bind to certain cell membrane-bound receptors, as well as in their soluble form (e.g., CD61, sCD61), thus modulating and regulating immune responses, and potentially the generation of protective antibodies [3]. Furthermore, the Small viral RNA segment of Andes virus (ANDV), Sin Nombre virus (SNV) and a few other OHV encodes a nonstructural protein that inhibits the interferon production in host cells, thus blunting immediate viral immunity and extending the asymptomatic incubation period [4, 3]. With respect specifically to ANDV and SNV, recent in vitro evidence has revealed remarkable selectivity, which remains under-studied in large part due to the scarcity of university-based BSL-4 facilities: namely, while ANDV appears to exhibit rather broad tropism, SNV seems to readily infect predominantly pulmonary endothelial cells. Perhaps directly consequential to this diversity in selectivity, ANDV infection appears to promote more widespread cell injury and inflammatory responses, including severely downregulating lipid metabolic pathways in lung epithelial cells [5]. It is now clear that the OHV family consists of at least seven genera and over 50 distinct species. OHV primarily infects small mammals, and predominantly wild rodents, usually without severe symptoms. Nonetheless, OHV can be zoonotic and a few members of the OHV family (e.g., SNV, ANDV) are now known to infect humans primarily via aerosols released from the infected rodents' urine and feces, and can cause serious and lethal kidney (i.e., hemorrhagic fever with renal syndrome, HFRS), and cardio-pulmonary disease (i.e., hantavirus cardiopulmonary syndrome or hantavirus pulmonary syndrome, HCPS or HPS) in humans. While ANVD is predominant in South America (viz, named Andes because first identified [1995] in the Andes regions of Argentina predominantly, and Chile), SNV is predominant in North America (1993), whereas the Puumala OHV (Finland, 1980) and the Dobrova OHV (Slovenia, 1992) are predominant in Europe. To this date, ANDV is the only OHV that manifests a human-to-human transmission, though both ANDV and SNV have a mortality rate estimated to 35-50% (i.e., one in three to one in two infected patients succumbs). OHV, and specifically SNV and ANDV - and particularly ANDV in light of the recent outbreak on cruise liner MV Hondius, which has left, to date, over a dozen confirmed cases, and 4 deaths - are now recognized of global public health significance [1, 2, 3, 4, 5, 6].

## OHV and Integrins:

As noted, the Gn and Gc glycoproteins form heterodimers that further assemble into tetrameric spikes on the OHV surface. These tetramers form the characteristic virions' regular geometric surface lattice, and allow binding to the host cells via cell membrane integrins, a family of transmembrane receptor proteins, usually composed of one of 18 alpha (α) and one of 8 beta (β) subunit, that bridge the extracellular matrix and the intracellular cytoskeleton. As adhesion molecules, integrins serve to anchor cells to the extracellular matrix, or in the case of OHV to anchor virions to the host cell membrane. As signaling molecules, integrins trigger intracellular signals that regulate cell survival, proliferation, and differentiation (viz, PTK2, etc.), as well as extracellular signals that the cell's affinity for ligands. As structural proteins, integrins promote cytoskeleton integrity via interactions with intracellular actin filaments, and adaptor proteins such as talin, vinculin, and α-actinin. Taken together, these multiple functions of integrins place integrins, and specifically αx/beta3(β3)-integrin (αx/CD61) at a critical point in cellular immune responses in general, viral immunity to OHV in particular [7, 8]. To be clear, since CD61 (viz, β3-integrin; 90-110 kDa) plays a critical role as an adhesive receptor in platelets and endothelial cells to regulate both platelets activation and vascular permeability, it is not surprising that OHV pathogenicity - both ANDV and SNV in humans - is dependent upon the virions' use of that receptor via their Gc-Gn tetrameric spike to adhere to, and eventually penetrate the host cell [8]. The genomic differences in Gc and Gn between ANDV and SNV probably contribute to the observed variabilities affinity to the CD61 ligand, thus efficiency in internalization, and consequently asymptomatic incubation period [8, 9]. While CD61 is predominantly a membrane-bound transmembrane receptor, it can be detected in a soluble form (sCD61) in blood serum and urine, a product of proteolytic shedding, often consequential to inflammation-related activation of matrix metalloproteinases, which then clip the extracellular domain of CD61 directly at the cell surface [10]. It is possible and even probable that sCD61 retains binding affinity for a variety of its ligands, including the Gc-Gn tetrameric spike of OHV, therefore putatively, sequestering circulating OHV before it can bind to its target cell and delaying the onset of productive infection. It would follow, therefore, in light of the observation cited earlier that ANDV might generate a broader and more widespread inflammatory response then SNV [5], that ANDV might also be characterized by a higher concentration of sCD61 than SNV at the early post-infection stages, which may contribute to the remarkably longer incubation period in ANDV (average 40+ days), compared to SNV (average 15-20 days).

## ANDV versus SNV:

Of course, what may also determine the distinctly different asymptomatic incubation period between ADNV and SNV may be intrinsic differences in Gc and Gn, which probably contribute to the observed variabilities affinity its to the CD61 ligand, thus efficiency in internalization, and eventually asymptomatic incubation period [8, 9]. In point of fact the structural blueprint of the ANDV spike reveals three principal molecular contacts among Gn1-Gc1, Gn1-Gc2 and Gn2-Gc1 that stabilize the tetrameric assembly, aid in pH sensing, and determine its interactions of specific neutralizing antibodies (e.g., ADI- 65534) [11]. By contrast, the Gc-Gn tetrameric spike structure of SNV interacts with a distinct neutralizing human monoclonal antibodies (e.g., SNV-42), which demonstrates its specificity with respect to the tetrameric assembly of ANDV [12].

## Interventions: 

To this date, there are no preventive anti-OHV, anti-ANDV or anti-SNV vaccine available. Nonetheless, Moderna is said to be developing a mRNA-based vaccine collaboration with the Korea University College of Medicine. Also, there is no anti-OHV antiviral treatment available. But, attempts have been conceptualized to treat patients with cocktails of existing drugs. Case in point, one of the passenger from the MV Hondius cruise ship - viz, 69-year old male, 71 kg, with localized prostate cancer treated with stereotactic body radiotherapy (last treatment, February 2026) and on the oral alpha-blocker for benign prostate hyperplasia containment, tamsulosin - positive for ANDV by RT-PCR though still asymptomatic, was treated experimentally with a mix of the guanine analog ribavirin (initially intravenous, then switched to oral on day +4), oral favipiravir, the synthetic pro-drug analog to guanosine and adenosine, subcutaneous administration of the bradykinin β2 receptor antagonist, icatibant and oral administration of the JAK inhibitor, baricitinib. The initial clinical profile and prognosis is promising, though final outcomes still pending [13]. In point of fact, the oral pro-drug favipiravir - i.e., converted by metabolism to its active form, favipiravir-RTP, after ingestion - is considered so promising in reducing the viral load by competitively inhibiting the viral RNA-dependent RNA polymerase enzyme, and thus stopping the virus from transcribing and replicating, that its principal maker worldwide, Fujifilm Pharmaceuticals, has preventively shipped 1,400 tablets to the European Medical Agency for distribution to individual EU member States [14]. In addition, interventions targeting tangential therapies directed at CD61, CD61-containing integrin complexes, or sCD61 are also under development, if anything as potential early biomarker of OHV infection antecedent to seropositivity. Systematic tests of administration of the integrin αIIb/β3 (CD61) antagonist, eptifibatide, usually given to block platelets from aggregating, and to prevent acute coronary syndrome, are in the final stages of planning [15, 16]. Related molecule inhibitors of integrin αx/CD61 are also being considered in that context [17].
